# Dynamic Mechanical Properties and Visco-Elastic Damage Constitutive Model of Freeze–Thawed Concrete

**DOI:** 10.3390/ma13184056

**Published:** 2020-09-12

**Authors:** Yan Li, Yue Zhai, Wenbiao Liang, Yubai Li, Qi Dong, Fandong Meng

**Affiliations:** 1 School of Geology Engineering and Geomatics, Chang’an University, Xi’an 710054, China; li.yu.bai@163.com (Y.L.); mengfandong@chd.edu.cn (F.M.); 2 School of Civil Engineering, Chang’an University, Xi’an 710061, China; hqlwb2007@126.com; 3 Shaanxi Science and Technology Holding Group, Xi’an 710077, China; dongqi922@126.com

**Keywords:** concrete, freeze–thaw cycle action, dynamic mechanical property, visco-elastic constitutive model, damage evolution

## Abstract

To study the dynamic mechanical characteristics and constitutive relation of concrete materials under freeze–thaw (FT) cycle conditions, C35 concrete was taken as the research object in this paper, and FT tests were carried out with a freeze–thaw range of −20–20 °C and a freeze–thaw frequency up to 50 times. By using the separated Hopkinson pressure bar (SHPB) system, impact compression tests of concrete specimens under different FT cycle actions were developed, then the dynamic fracture morphology, fracture block distribution, stress–strain curve, peak stress and other dynamic mechanical properties of concrete were analyzed, and the influence law of FT action and strain rate was obtained. Through introducing the freeze–thaw deterioration damage factor and the stress damage variable, the dynamic visco-elastic damage constitutive equation of freeze–thawed concrete was constructed based on component combination theory. Furthermore, the damage evolution process and mechanism of freeze–thawed concrete materials were revealed. The research results show that the dynamic mechanical properties of concrete under a freeze–thaw environment are the combined results of the freeze–thaw deterioration effect and the strain rate strengthening effect. The dynamic visco-elastic damage constitutive model established in this paper can effectively describe the dynamic mechanical properties of freeze–thawed concrete, and has the characteristics of few parameters and good effect. The stress damage evolution path of concrete goes backward with the increase of FT cycles and the development speed gradually slows down. The greater the difference in FT cycles, the greater the difference in stress damage path.

## 1. Introduction

The cold areas in China are widely distributed, and the seasonal and permanent cold areas account for more than three quarters of China’s land area. In recent years, with the rapid development of the economy, there are more and more projects built in the cold region, such as the Qinghai–Tibet Railway, the Sichuan–Tibet Railway, the China–Russia and China–Kazakhstan Oil Projects, as well as many long tunnels and dams. As a kind of material widely used in railway, highway, dam, tunnel and other cold region engineering, concrete will suffer from freeze–thaw damage in the long service of freeze–thaw environment. Under the action of the freeze–thaw cycle, the residual moisture in the interior pores will continuously freeze and thaw, and then cause the continuous development of the internal pores, the performance of the concrete structure will be greatly affected, easily causing structure cracking, deformation and even instability failure [1,2]. Therefore, in cold regions, concrete structural deterioration due to freeze–thaw damage is a common problem. In recent years, many researchers have studied the freeze–thaw resistance of concrete [3,4,5,6,7] and established corresponding freeze–thaw damage models [8,9]. For example, Petros Petrounias [3,4] and Wojciech Piasta et al. [5] studied the effect of coarse aggregate performance on concrete under ordinary temperature and freeze–thaw conditions, respectively, and the results showed that the resistance of the aggregate to freezing and thawing was demonstrated to agree with the values of crushing resistance and the lowest contents of pores with diameters unsafe in terms of the freeze–thaw resistance. Bahram M. Taheri et al. [6] evaluated the freeze–thaw durability of pervious concrete specimens using the JC446-91 test method and revealed that the conditions and number of cycles used in JC446-91 were inadequate to evaluate the freeze–thaw durability of pervious concrete, especially for strong mixes. Rong-xin Peng et al. [7] proposed a meso-numerical simulation method to analyze the mechanical properties of freeze–thaw concrete, and the simulation model can effectively describe the stress–strain evolution behavior of freeze–thaw concrete. Ben Li et al. [8] developed an innovative mesoscopic damage parameter by following a unique calculation procedure in which the damage parameter is cumulatively computed from the minimum to the maximum by iterative cycles instead of using a direct calculation method. Sun Ming et al. [9] proposed a cohesion reduction parameter and investigated the mechanism of damage evolution and plasticity development of concrete materials subjected to freeze–thaw cycles during the load process.

The above studies on the mechanical properties of freezing-thawing concrete are of great significance for guiding the prevention of freezing-thawing injury in civil engineering in cold regions. However, most of them focus on the static mechanical properties. Studies have shown that [10,11] concrete projects in cold areas are often subjected to dynamic loads during construction and use, such as excavation blasting load, and impact load caused by vehicles, waves and earthquakes. The study of the dynamic mechanical properties and constitutive relation of freeze–thawed concrete is of important engineering and theoretical significance, and can provide experimental data and a theoretical basis for the mechanism and prediction of freeze–thaw disasters.

The constitutive model is one of the important bases on which to study the strength and deformation of concrete, and reflects the stress–strain curve, the most basic mechanical property of concrete [12]. The component model is often used to study the dynamic constitutive relation of concrete. It is composed of the components in series or in parallel, and it simulates the actual stress–strain relationship by adjusting the parameters and the number of combined components, so that the stress–strain curve of the model is consistent with the test results. Component models established in this way are one-dimensional models, such as the Bingham Model, the Elastoplastic Model, the Maxwell Model, the Merchant Model, and so on. To better fit the test results, some models use multiple components. Although this approach achieves a good fitting effect, it increases the number of parameters, such as in the Zhu-Wang-Tang (Z-W-T) Model [13,14] and the various modified Z-W-T models [15,16]. The Z-W-T model is a nonlinear viscoelastic constitutive model that is based on the Green–Rivlin multi-integral nonlinear constitutive theory and is suitable for a strain rate of 10^−4^–10^3^ s^−1^. Due to the good fitting accuracy, the Z-W-T model has been widely used in simulating constitutive relations of brittle materials such as concrete and rock [15,16]. Nevertheless, its expression is relatively complex, and the number of fitting parameters is relatively large. Therefore, the constitutive model with good prediction accuracy and simple expression has become the research target of many researchers.

Directed at C35 concrete suffering from different numbers of freeze–thaw cycles (0, 10, 20, 30, 40, 50), a series of impact compression tests was carried out on a Φ50 mm (i.e., the diameter of the bar is 50 mm) separated Hopkinson pressure bar (SHPB) system in this paper. The dynamic fracture morphology, fracture block distribution, stress–strain curve, peak stress and other dynamic mechanical properties of concrete were analyzed, and the influence law of freeze–thaw cycle and strain rate was obtained. By introducing the freeze–thaw deterioration damage factor and the stress damage variable, the dynamic visco-elastic damage constitutive equation of freeze–thawed concrete was constructed based on component combination theory. The damage evolution process and mechanism of freeze–thawed concrete materials were revealed.

## 2. Test Design

### 2.1. Specimen Preparation

C35 concrete was applied in this experiment, the proportion of the basic materials of Portland cement, stone, sand, water, fly ash, and water reducing agent was 1:3.80:2.03:0.56:0.40:0.02. The Portland cement was P.0.42.5 ordinary Portland cement produced by Shaanxi Qinling Cement Building Materials Limited Liability Company, with a density of 3.05 g/cm^3^, an initial setting time of 208 min, a final setting time of 260 min, a bending strength of 8.6 MPa, a compressive strength of 51 MPa, and the volume stability was qualified. The coarse aggregate was 5–16 mm continuously graded gravel. The fine aggregate was continuously graded medium river sand, with a fineness modulus of 2.42. Before the concrete was prepared, the soil in the river sand was washed clean and the sand was then placed in a drying box to dry for 24 h. The water was laboratory tap water and conformed to the specification requirements. Fly ash was secondary ash, fineness 43 μm, density 2.4 g/cm^3^, moisture content about 5%. The pH value of the water-reducing agent was 7–9, the water-reducing rate was about 20%–35%, and the solution was prepared with a concentration of 26%–28%.

The mixture was placed into a 100 mm × 100 mm × 100 mm steel mold, and compacted on the shaking table. After 24 h, the mold was removed for 28 days of curing in the standard curing room. Through coring and cutting, the specimens were processed into cylinders with a diameter of 50 mm and a height of 25 mm. Then the two end faces of the specimen were polished to ensure that the non-parallelism and non-perpendicularity were both less than 0.02 mm [17]. On this basis, the specimens were numbered, measured in height, diameter and mass, and then put into a drying box for 48 h, and then into a saturation dish. Distilled water was injected into the saturation dish, and the air was pumped continuously at a pressure of 0.1 MPa for 4 h until no bubbles spilled onto the surface of the specimens; they were then soaked for more than 24 h to achieve saturation of the specimens.

### 2.2. Freeze–Thaw Cycle Test

According to the “standard for test methods of long-term performance and durability of ordinary concrete (GB/T 50082-2009)”, frost resistance tests for concrete include the fast freezing method and the slow freezing method. The slow freezing method is suitable for the determination of concrete specimens under conditions of gas-freeze-water thawing. The fast freezing method is suitable for speciments under conditions of water-freeze-water thawing.

The FT cycle test in this paper adopted the slow freezing method and was performed in an automatic low-temperature freeze–thaw chamber (model: LD-1; temperature measurement accuracy: ±0.5 °C; temperature range: 50–−40 °C, produced by Xi’an Yaxing Civil Instrument Co. Ltd., Xi’an, China). According to clause 4.1.4 of the “Standard for test methods of long-term performance and durability of ordinary concrete (GB/T 50082–2009)”, “the time required from the time the specimen is installed to the time when the temperature drops to −18 °C should be within (1.5–2.0) h, and the temperature in the freezing-thawing chamber shall be maintained at (−20–−18) °C during freezing”, “the freezing time of each FT cycle should not be less than 4h”, “Immediately after the freezing, water with a temperature of (18–20) °C should be added, and the melting time should not be less than 4 h. After thawing, it is deemed that this freeze–thaw cycle has ended, and the next freeze–thaw cycle can be started”.

**Figure 1:**
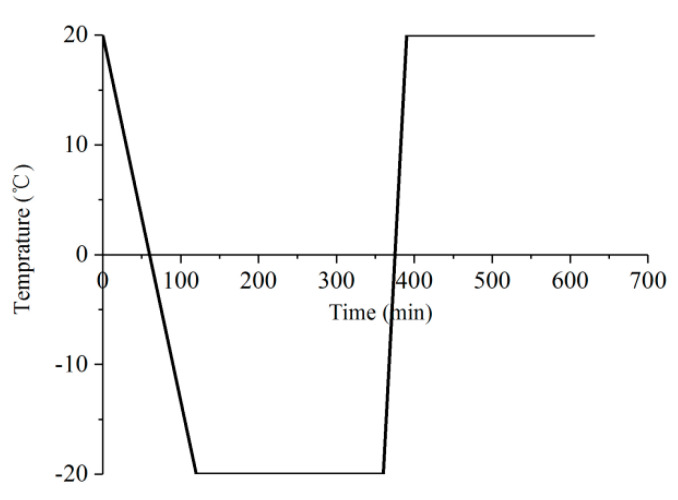
Time-history curve of one FT cycle.

Considering the above standard requirements, the temperature range and cooling rates were determined as follows. The freeze–thaw temperature variation range was −20–+20 °C, and freeze–thaw cycles were carried out 0, 10, 20, 30, 40 and 50 times, respectively. The time-history curve of one FT cycle is shown in Figure 1, showing that the temperature was slowly decreased from 20 to −20 °C (this process lasted for 120 min), this temperature was maintained constant for 240 min, then the temperature was rapidly increased to room temperature at 20 °C (this process lasted for 30 min), and then this temperature was maintained constant for 240 min. The total time for one FT cycle was 630 min.

### 2.3. Impact Compression Test

**Figure 2:**
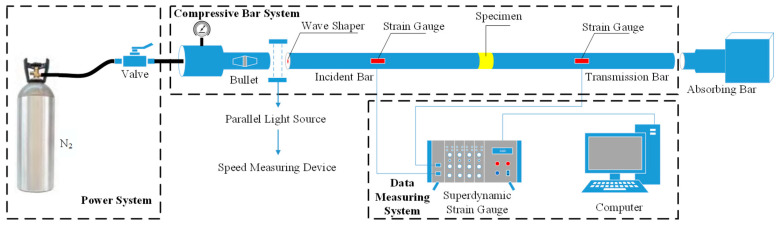
Diagram of SHPB system.

Aiming at the concrete suffering from different numbers of FT cycles (0, 10, 20, 30, 40, 50), uniaxial impact compression tests under different strain rates were carried out on a Φ50 mm SHPB system (custom-built device, Figure 2).

To reduce the dispersion effect caused by transverse inertial motion of the bar particles, and to extend the time before the incident pulse reached the peak value, so as to ensure that the specimen had enough time to reach uniform stress before failure, waveform shaping should be considered [18]. In this paper, a large number of tests were conducted on red copper sheets of different sizes, and the waveform effects after shaping were compared and analyzed. When the impact velocity was 5.4, 8.8 and 11.3 m/s, the thickness of the shaping sheets was determined to be 1 mm, and the diameter was 25, 20 and 15 mm, respectively. During the SHPB test, the two end faces of the specimen and the contact surface between the SHPB system and the specimen were uniformly daubed with molybdenum disulfide to reduce the friction force. When loading, the valve switch was manually controlled.

## 3. Analysis of Test Results

### 3.1. Dynamic Fracture Morphology

**Figure 3:**
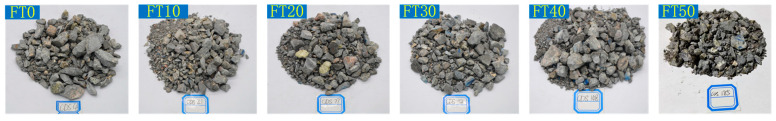
Dynamic fracture morphology of concrete under different numbers of freeze–thaw cycles.

**Figure 4:**
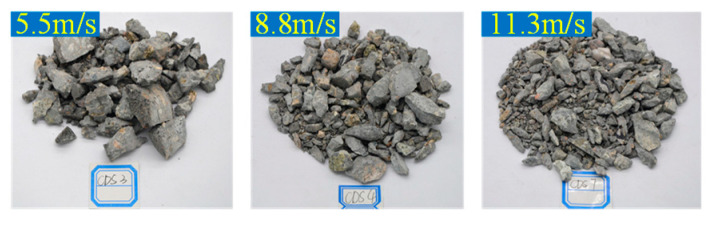
Dynamic fracture morphology of concrete under different impact velocities.

Taking the 8.8 m/s condition as an example, the dynamic fracture morphology of concrete under different numbers of FT cycles is shown in Figure 3. Taking the FT0 as an example, the dynamic fracture morphology of concrete under different impact velocities is shown in Figure 4.

It can be seen from Figure 3 and Figure 4 that, when the number of FT cycles was less or the impact velocity was lower, the dynamic compression failure of concrete specimens was in the form of large or medium grain size fragments in strips and blocks. For example, under the conditions of FT0 and 5.4 m/s. With the increase of FT cycles and impact velocity, the concrete specimens were more and more seriously broken, and most of the fragments were uniform fine particles. For example, under the conditions of FT50 and 11.3 m/s. This is because when the number of FT cycles is less or the impact velocity is lower, the internal cracks of the specimens are fewer, and the damage is lighter. When the dynamic load was applied, the crack in the specimen extended directly through in situ, leading to a small degree of fracture and a split failure mode. When the number of FT cycles increased or the impact velocity improved, the distribution density of axial and transverse cracks grew, resulting in more serious damage. Moreover, when applying dynamic load, the specimen absorbed energy and produced more small cracks, which quickly became unstable, and “cut” the specimen into small particles, resulting in serious crushing degree and a crushing failure mode.

### 3.2. Fragmentation Distribution and Fractal Characteristics

**Figure 5:**
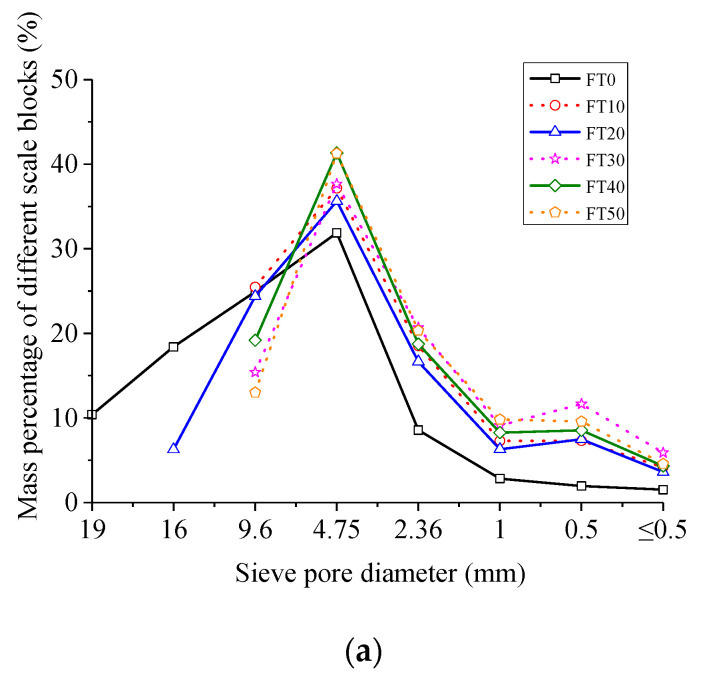

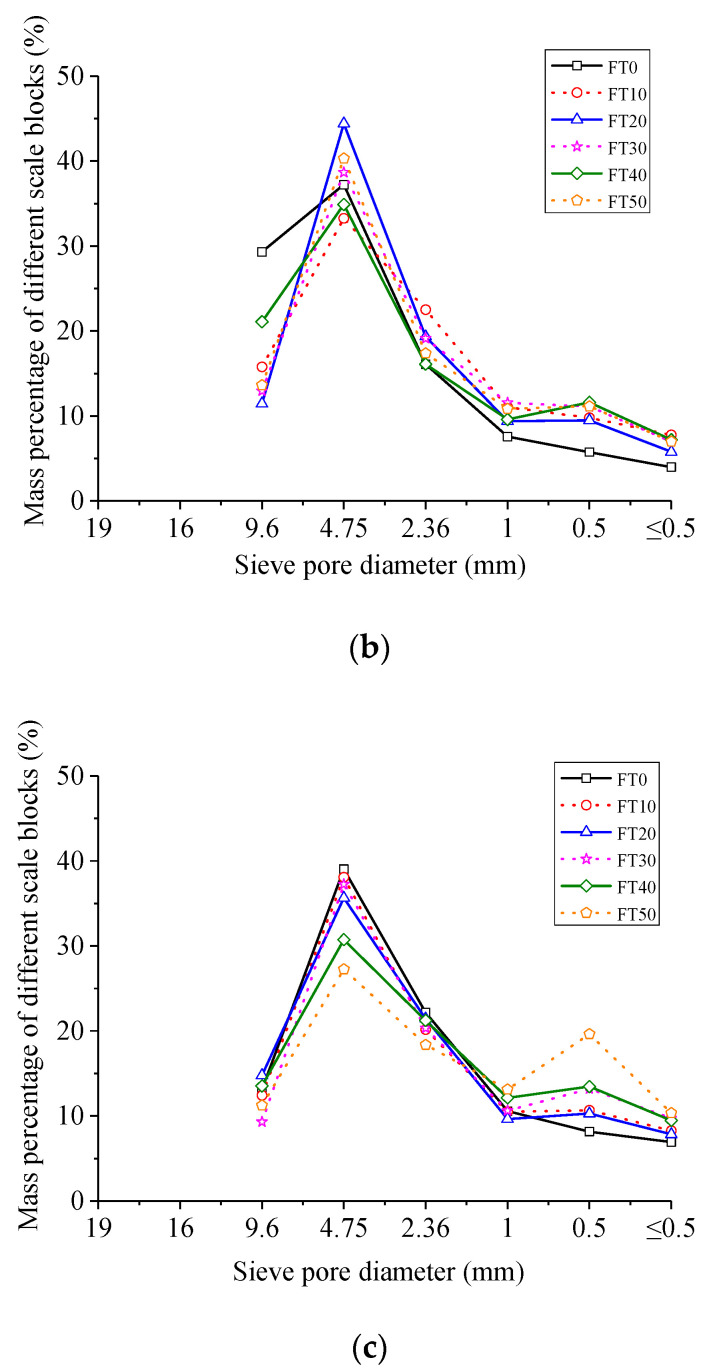
Scale–mass distribution law of concrete fragments. (**a**) 5.4 m/s; (**b**) 8.8 m/s; (**c**) 11.3 m/s.

After the impact compression test, the concrete fragments were collected and screened statistically by the ZBSX-92A standard vibration pendulum (produced by Xingye Test Instrument Co., Ltd., Cangzhou, China). The diameter of the sieve holes was 0.5, 1, 2.36, 4.75, 9.6, 16 and 19 mm, respectively. After the screening test, the quality of the subscreen of each stage was weighed by a high-sensitivity electronic scale. The scale–mass distribution law of concrete fragments under different conditions is shown in Figure 5.

As can be seen from Figure 5, the greater the number of FT cycles or the higher the impact velocity, the greater the mass proportion of small and medium-sized broken blocks. For example, when the impact velocity was 5.4 m/s, under the conditions of FT0 and FT50, the mass percentage of particle size greater than or equal to 9.6 mm was 53.73% and 13.00%, respectively, and that less than or equal to 4.75 mm was 46.75% and 85.56%, respectively. When the FT cycle was 0 and the impact velocity was 5.4, 8.8 and 11.3 m/s, the mass percentage of particle size greater than or equal to 9.6 mm was 53.73%, 29.32% and 13.14%, respectively, and that less than or equal to 4.75 mm was 46.75%, 70.64% and 86.84%, respectively. This was because the greater the number of FT cycles, the more serious the concrete degradation, and the higher the impact velocity, the greater the impingement energy, resulting in smaller specimen crushing size.

According to the mass–frequency relationship [19], the distribution equation of concrete fragments under the impact loading was

(1) $$Y=\frac{M_{r}}{M_{T}}={\left(\frac{r}{r_{m}}\right)}^{3-\chi }$$

where r, $r_{m}$ and χ are the particle size, the maximum size and the fractal dimension of crushing blocks, respectively; $M_{r}$ is the total mass of crushing blocks with particle size smaller than r; and $M_{T}$ is the total mass of specimen crushing blocks.

The natural logarithm on the both sides of Equation (1) is taken to get

(2) $$\ln Y=\ln\left(\frac{M_{r}}{M_{T}}\right)=\left(3-\chi \right)\ln\left(\frac{r}{r_{m}}\right)$$

In the ln(*M_r_*/*M_T_*)~ln*r* coordinate system, the slope of the fitting line was K=3−χ. Therefore, the fractal dimension of the crushing blocks can be calculated using the mass-granularity method [20], expressed as

(3) χ=3−K

**Figure 6:**
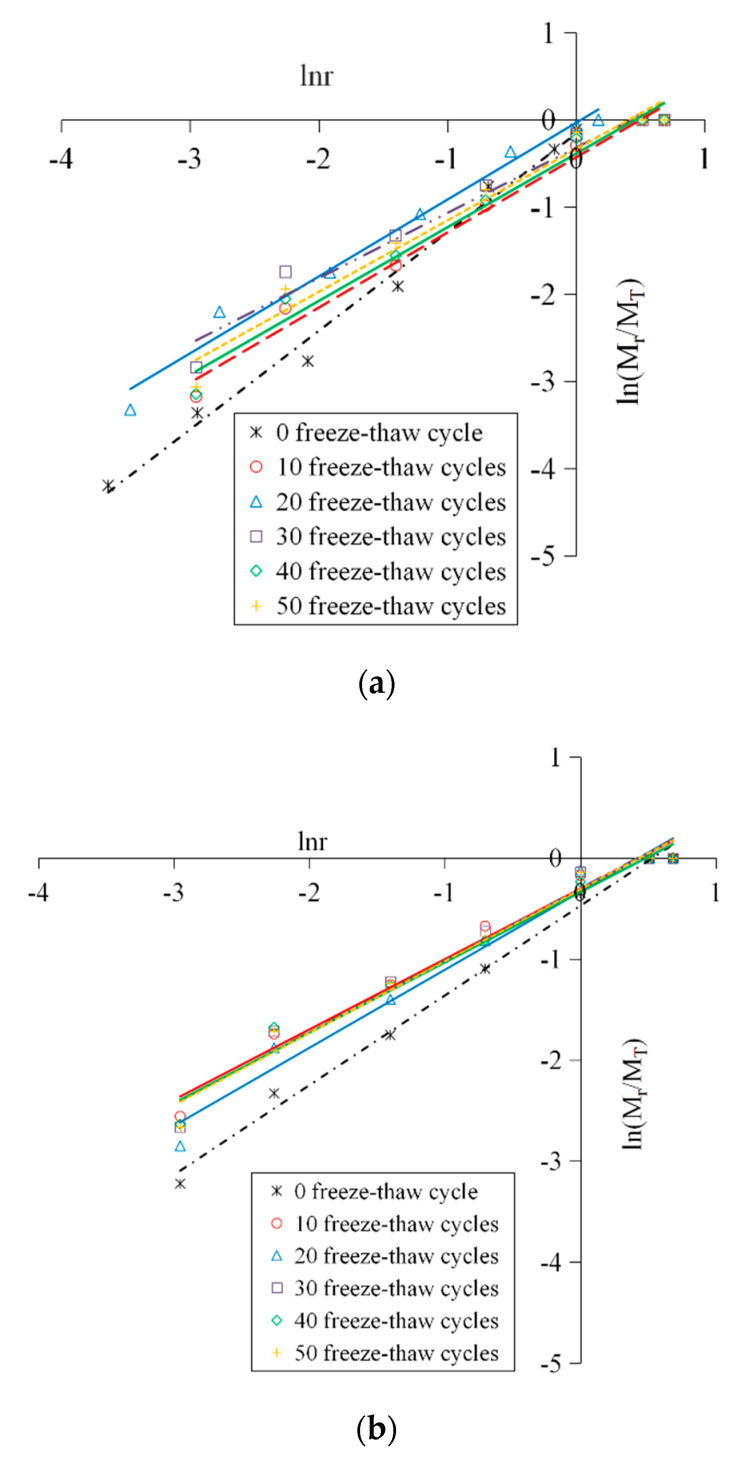

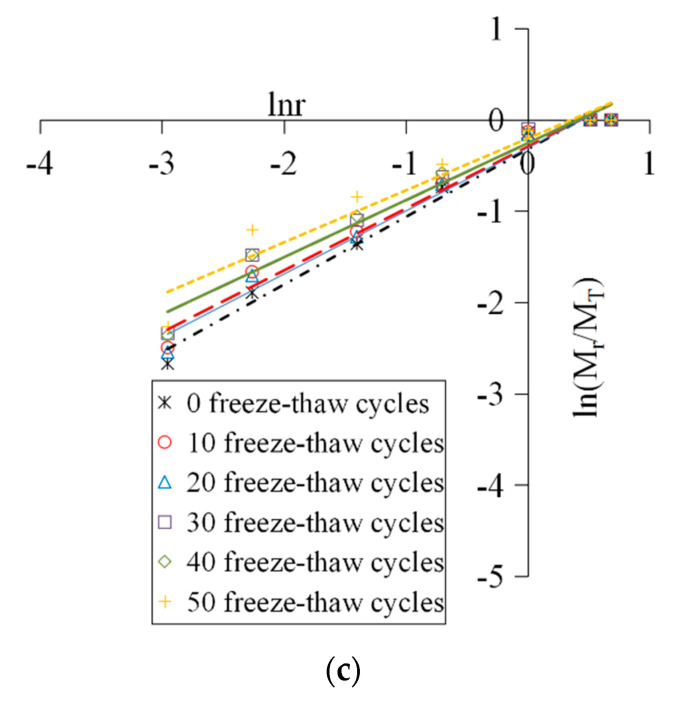
Logarithmic curves of the concrete crushing blocks. (**a**) 5.4 m/s; (**b**) 8.8 m/s; (**c**) 11.3 m/s.

The logarithmic curves of the concrete crushing blocks under different conditions are shown in Figure 6, and the fractal dimensions of the crushing blocks were computed as shown as Table 1.

It can be found in Figure 6 that all data points under different working conditions showed a good linear correlation in the ln(*M_r_*/*M_T_*)−ln*r* coordinate system, indicating that the distribution of the concrete fragments had fractal characteristics. This was because the distribution of the mesoscopic cracks and pores inside the concrete conformed to the fractal theory, and had self-similarity under different scales. The freeze–thaw deterioration and the impact crushing process were the direct results of crack extension; therefore, the crushing blocks of the specimens subjected different numbers of freeze–thaw cycles and different levels of impact loading also showed a certain self-similarity, that is, they satisfied the power law characteristics, and it was a fractal of statistical significance. In summary, the concrete material had fractal properties from the microscopic damage to the macroscopic fracture, and the smaller the fragment size, and the more serious the material breakage.

It can be drawn from Table 1 that, when under a certain loading rate, the more FT cycles the concrete had suffered, the lager the fractal dimension of the crushing blocks was and the smaller the dynamic peak stress was. In addition, under the same FT cycle conditions, although the fractal dimension increased with the increase of loading speed due to the obvious rate correlation of concrete dynamic strength, the dynamic peak stress nevertheless tended to increase.

### 3.3. Dynamic Stress–Strain Curve

**Figure 7:**
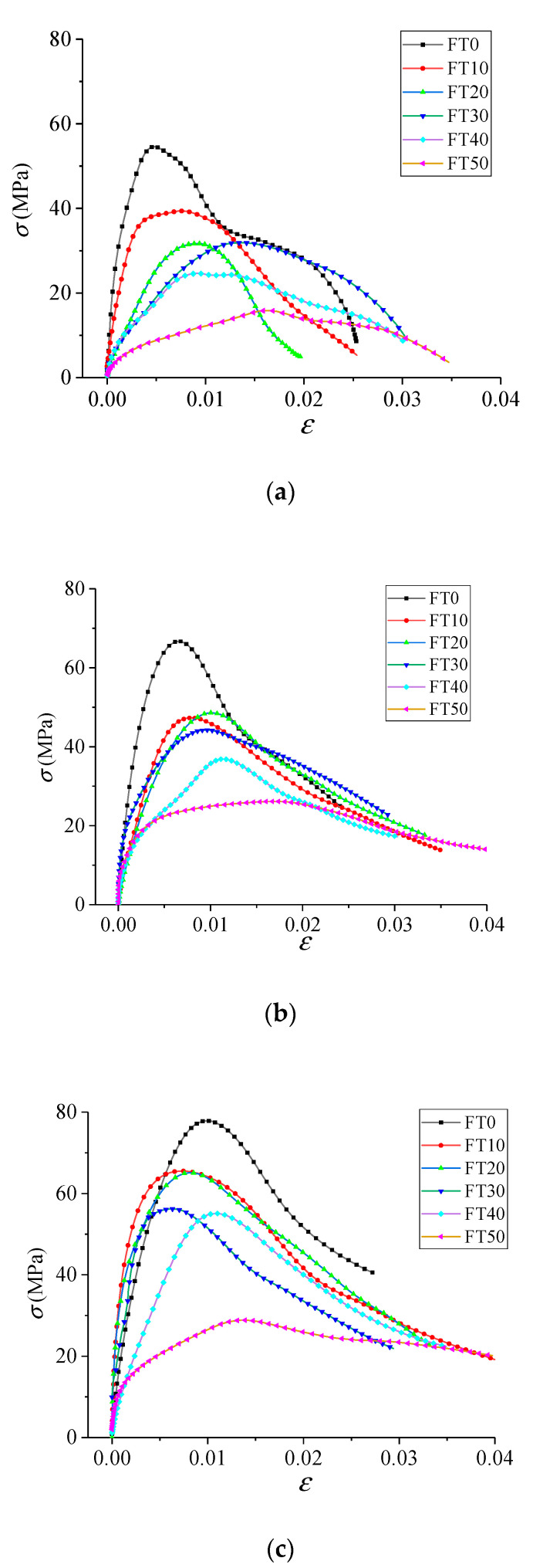
Dynamic stress–strain curves of freeze–thawed concrete. (**a**) 5.4 m/s; (**b**) 8.8 m/s; (**c**) 11.3 m/s.

The dynamic stress–strain curves of the freeze–thawed concrete are shown in Figure 7.

It can be drawn from Figure 7 that the freeze–thaw action has a significant deterioration effect on the dynamic mechanical properties of concrete. For example, when the impact velocity was 5.4 m/s, the peak stress was 55.10 and 16.29 MPa, respectively, under the conditions FT0 and FT50, which corresponds to a reduction of 70.44%. When the impact velocity was 11.3 m/s, the peak stress was 79.05 and 29.70 MPa, respectively, under the conditions FT0 and FT50, which corresponds to a decrease of 62.43%. This is because under the action of freeze–thaw cycle, the water inside the concrete freezes and expands, resulting in internal stress. When the internal stress exceeds the maximum allowable value of concrete, pores and micro-cracks in concrete will gradually generate, increase, expand and connect with each other, resulting in an increase in damage degree and a decrease in dynamic strength in macroscopic mechanical properties. This conclusion is consistent with that of the reference [21].

By comparing Figure 7a–c, it can be found that the concrete material showed an obvious strengthening effect of strain rate. For example, under the condition FT0, when the impact velocity was 5.4 m/s, the peak stress was 55.10 MPa, and when the impact velocity was 8.8 and 11.3 m/s, the peak stress was 68.03 and 79.05 MPa, corresponding to increases of 23.47% and 43.47%, respectively. Under the condition FT50, when the impact velocity was 5.4 m/s, the peak stress was 16.29 MPa, and when the impact velocity was 8.8 and 11.3 m/s, the peak stress was 27.31 and 29.70 MPa, corresponding to increases of 67.65% and 82.32%, respectively. This is because the larger the initial impact velocity is, the higher the impact energy the specimen absorbs in a short time, and the internal damage of the specimen will not have enough time to fully develop and penetrate, so the macro mechanical property was relatively high.

In conclusion, the dynamic mechanical properties of concrete under a freeze–thaw environment are a result of the combination of freeze–thaw deterioration and strain rate enhancement.

### 3.4. Quantitative Analysis of Freeze–Thaw Deterioration Effect and Strain Rate Enhancement Effect

Dynamic peak stress, the basic mechanical parameter, is taken as a characteristic index, and the relative loss of peak stress is defined as the freeze–thaw degradation effect factor; then

(4) $$\xi =1-\frac{\sigma_{\mathrm{FT}i}}{\sigma_{\mathrm{FT}0}}$$

where, ξ is the freeze–thaw degradation effect factor, reflecting the influence of freeze–thaw action on the dynamic peak stress; $\sigma_{\mathrm{FT}i}$ is the peak stress of concrete under uniaxial impact after different FT cycles; $\sigma_{\mathrm{FT}0}$ is the peak stress of concrete under uniaxial impact under FT0.

**Figure 8:**
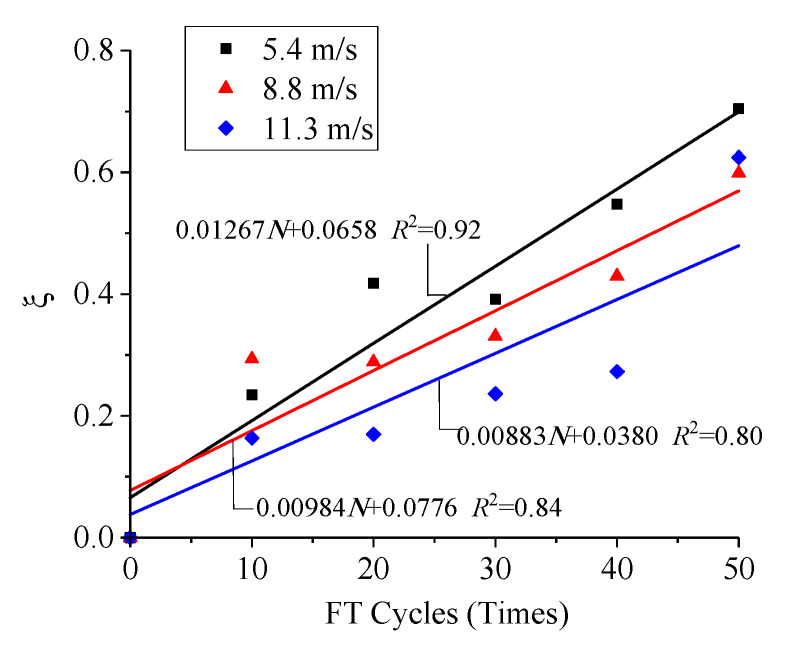
Change laws of ξ with FT cycles.

Change laws of the freeze–thaw degradation effect factor ξ with the FT cycles are shown in Figure 8.

Figure 8 shows that the freeze–thaw degradation effect factor at different impact velocities has similar characteristics under changing numbers of FT cycles, and these approximately satisfy a linear relationship

(5) ξ=aN+b

where *N* is the number of FT cycles, and *a* and *b* are fitting parameters. By statistical regression, the fitting correlations were 0.92, 0.84 and 0.80, respectively, under different loading conditions, suggesting that Formula (5) could better reflect the changing law of the freeze–thaw degradation effect factor with the FT cycles.

Figure 8 also indicates that, because of the FT degradation effect, ξ increases with the increase in the number of FT cycles. In addition, due to the coupling effect of strain rate enhancement, ξ decreases with the increase of impact velocity under the same FT conditions.

**Figure 9:**
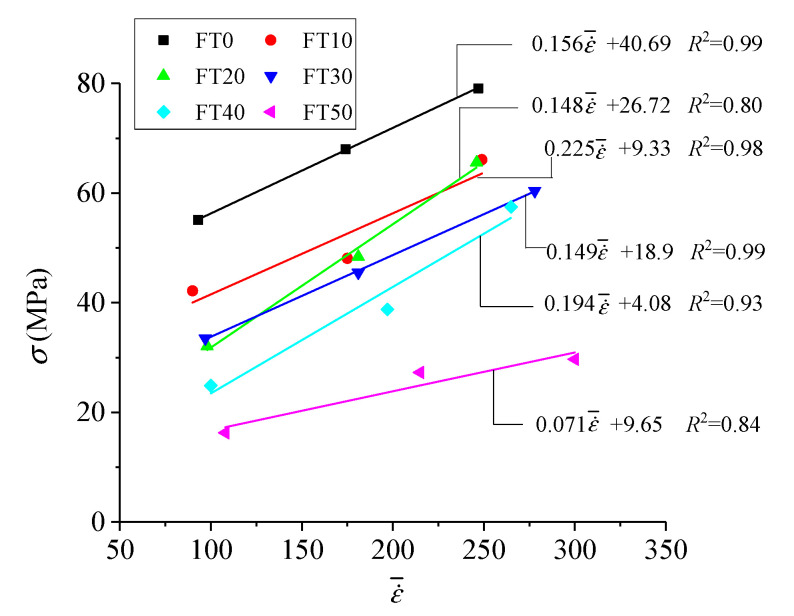
Variation rules of dynamic peak stress with strain rate.

Variation rules of the dynamic peak stress of freeze–thawed concrete with strain rate are shown in Figure 9.

Figure 9 shows that the dynamic peak stress and strain rate satisfy a linear relationship $\sigma_{\mathrm{FT}i}=c\bar{\dot{\varepsilon }}+d$, where $\bar{\dot{\varepsilon }}$ is the average strain rate, and *c* and *d* are fitting parameters. When the FT cycles were 0, 10, 20, 30, 40 and 50, the fitting correlations were 0.99, 0.80, 0.98, 0.99, 0.93 and 0.84, respectively, which indicated that the linear expression could well reflect the change rules of dynamic peak stress with strain rate.

Figure 9 further shows the rate correlation of the dynamic peak stress of concrete. It is suggested that, in contrast to static mechanical properties, the influence of strain rate on the dynamic peak stress of concrete cannot be ignored, and must be considered when constructing a constitutive model.

## 4. Dynamic Visco-Elastic Damage Constitutive Model

### 4.1. Construction Method of the Constitutive Equation

As the internal structure of concrete is not homogeneous, and contains many defects, the strength of each micro-element is not the same. For the convenience of study, and considering the continuity of concrete damage in the loading process, it is assumed that the concrete is divided into several micro-elements with different defects. The micro-elements are divided into small ones, and meet the duality of size, that is, on the one hand, their size is large enough to contain enough micro-joints, micro-cracks and other micro-information from the microscopic perspective, while on the other hand, from the macroscopic point of view, their size is small enough to be considered as a particle of continuous damage mechanics.

The test results showed that the dynamic mechanical properties of freeze–thawed concrete mainly reflected the evolution process of micro-cracks under the thermal and force coupling action. That is to say, the dynamic mechanical properties of freeze–thawed concrete are the composite damage evolution of freeze–thaw fracture and stress-damage fracture, and the stress–strain curve will show certain differences when the freeze–thaw action or strain rate is changed. Therefore, it is necessary to reflect the above basic physical facts reasonably and appropriately in order to establish the dynamic constitutive equation.

In this paper, freeze–thaw degradation effect factor ξ was adopted to consider the effect of freeze–thaw degradation. Based on the classical Hooke law, damage variable was introduced to describe the growth of micro-cracks, namely the degradation caused by stress damage fracture. Then the stress–strain equation of freeze–thawed concrete can be expressed as

(6) $$\sigma =\left(1-\xi \right)(1-D)E_{0}\varepsilon$$

where $E_{0}$ was the initial elastic modulus of concrete; *D* was damage variable, 0≤D≤1.

On this basis, according to the component combination theory, concrete can be regarded as a union of a damaged body and a viscous body, and the influence of the strain rate can be reflected through the viscous body. Finally, the dynamic damage constitutive model of freeze–thaw concrete was derived.

### 4.2. Damage Variable

Concrete is obviously a brittle material under freeze–thaw action and impact load, and its plastic strain is very small. Therefore, the plastic deformation part can be ignored [22]. The concrete damage is continuous during the loading process. It was assumed that the strength of each micro-element was subject to probability distribution [12], and the damage variable *D* had the following relationship with φ(ε)

(7) $$\frac{dD}{d\varepsilon }=\varphi (\varepsilon )$$

Studies have shown that [22,23,24,25,26,27] Weibull distribution is particularly suitable for describing the fracture process of materials such as concrete and rock, then

(8) $$\varphi (\varepsilon )=\frac{m}{\alpha }{\left(\varepsilon -\gamma \right)}^{m-1}\exp[-\frac{{\left(\varepsilon -\gamma \right)}^{m}}{\alpha }]$$

where α and m are the scale parameters and shape parameters, respectively. γ is the position parameter, which was the damage threshold. Based on Formulas (7) and (8), the damage variable *D* was

(9) $$D=\int_{\gamma }^{\varepsilon }\varphi (x)dx=\frac{m}{\alpha }\int_{\gamma }^{\varepsilon }{\left(x-\gamma \right)}^{m-1}\exp[-\frac{{\left(x-\gamma \right)}^{m}}{\alpha }]dx{=-\exp[-\frac{{\left(x-\gamma \right)}^{m}}{\alpha }|}_{\gamma }^{\varepsilon }=1-\exp[-\frac{{\left(\varepsilon -\gamma \right)}^{m}}{\alpha }]$$

As concrete is a kind of multiphase composite material, it contains many micro-cracks and micro-defects. In other words, it has initial damage. Therefore, the damage threshold value was set as 0 in this paper, namely γ=0. Therefore, the damage variable *D* can be further simplified as

(10) $$D=1-\exp[-\frac{\varepsilon^{m}}{\alpha }]$$

### 4.3. Dynamic Visco-Elastic Damage Constitutive Model Based on Component Combination Theory

**Figure 10:**
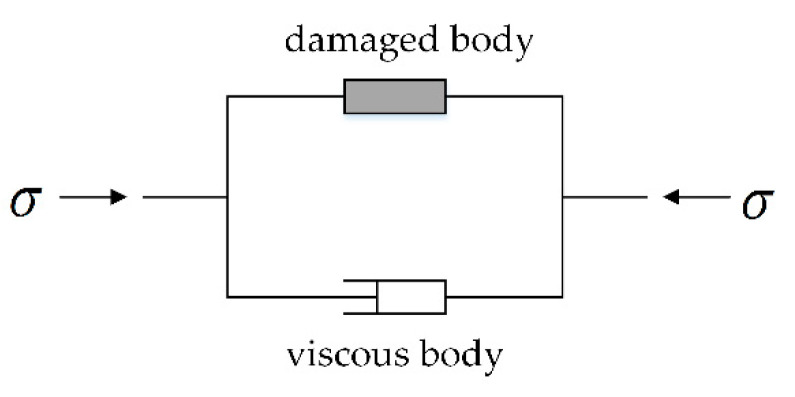
Visco-elastic damage model.

Because concrete has properties of both viscous liquid and statistical damage, it can be regarded as a combination of the parallel connections of a viscous body and a damaged body [28], as shown in Figure 10.

Based on the component combination theory

(11) $$\sigma =\left(1-\xi \right)(1-D)E_{0}\varepsilon +\eta \dot{\varepsilon }=\left(1-\xi \right)\exp[-\frac{\varepsilon^{m}}{\alpha }]E_{0}\varepsilon +\eta \dot{\varepsilon }$$

Equation (11) is the dynamic visco-elastic damage constitutive equation of freeze–thawed concrete. The unknown parameters in the equation can be obtained by inversion analysis based on the test results.

### 4.4. Z-W-T Model

**Figure 11:**
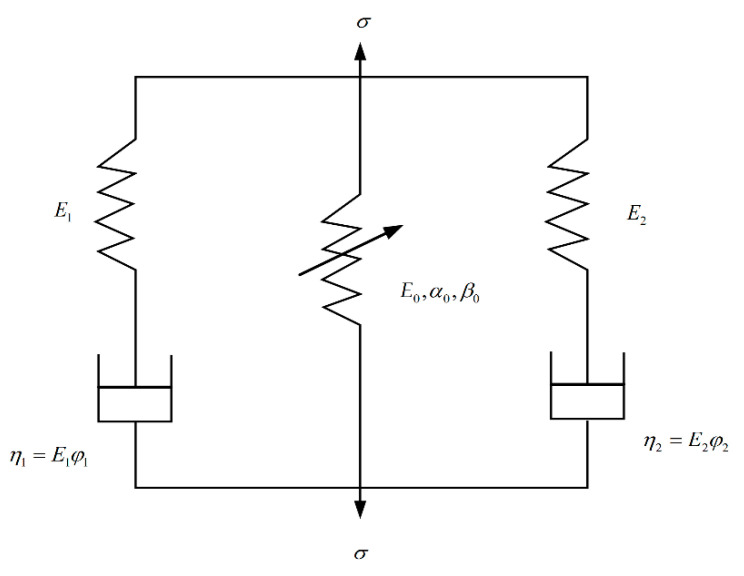
Z-W-T model.

The Z-W-T model is shown in Figure 11 [13,14].

The Z-W-T model is composed of a nonlinear elastomer in parallel with two Maxwell models. The first Maxwell model ($E_{1}$,$\varphi_{1}$) describes the visco-elastic response under quasi-static conditions and low strain rates, and the second Maxwell model ($E_{2}$,$\varphi_{2}$) describes the visco-elastic response under high strain rates. The expression of the Z-W-T model is

(12) $$\sigma =E_{0}\varepsilon +\alpha_{0}\varepsilon^{2}+\beta_{0}\varepsilon^{2}+E_{{}^{1}}\int_{0}^{t}\varepsilon (\tau )\exp(-\frac{t-\tau }{\varphi_{1}})d\tau +E_{{}^{2}}\int_{0}^{t}\varepsilon (\tau )\exp(-\frac{t-\tau }{\varphi_{2}})d\tau$$

where $E_{0}$, $E_{1}$, $\varphi_{1}$, $E_{2}$, $\varphi_{2}$ are material constants, $E_{0}$,$\alpha_{0}$,$\beta_{0}$ are elastic constants, $E_{1}$,$E_{2}$ are linear elastic modulus, and $\varphi_{1}$,$\varphi_{2}$ are relaxation time.

In the SHPB test, the deformation of concrete is very small, and the stress–strain curve in the quasi-static test is approximately linear. Therefore, the first part of nonlinear elasticity in Formula (12) can be regarded as linear elasticity, that is, Formula (12) can only take the first term. The SHPB test can be regarded as an approximately constant strain rate loading process. Therefore, the Z-W-T model expression can be written as follows

(13) $$\sigma =E_{0}\varepsilon +E_{{}^{1}}\varphi_{1}\dot{\varepsilon }(1-e^{-\frac{\varepsilon }{\dot{\varepsilon }\varphi_{1}}})+E_{2}\varphi_{2}\dot{\varepsilon }(1-e^{-\frac{\varepsilon }{\dot{\varepsilon }\varphi_{2}}})$$

Furthermore, the Z-W-T model expression after considering the damage was

(14) $$\begin{array}{c} \sigma =\left(1-D\right)\left[E_{0}\varepsilon +E_{{}^{1}}\varphi_{1}\dot{\varepsilon }(1-e^{-\frac{\varepsilon }{\dot{\varepsilon }\varphi_{1}}})+E_{2}\varphi_{2}\dot{\varepsilon }(1-e^{-\frac{\varepsilon }{\dot{\varepsilon }\varphi_{2}}})\right]\\ =\exp[-\frac{\varepsilon^{m}}{\alpha }]\left[E_{0}\varepsilon +E_{{}^{1}}\varphi_{1}\dot{\varepsilon }(1-e^{-\frac{\varepsilon }{\dot{\varepsilon }\varphi_{1}}})+E_{2}\varphi_{2}\dot{\varepsilon }(1-e^{-\frac{\varepsilon }{\dot{\varepsilon }\varphi_{2}}})\right] \end{array}$$

### 4.5. Validation Analysis of Suitability

**Figure 12:**
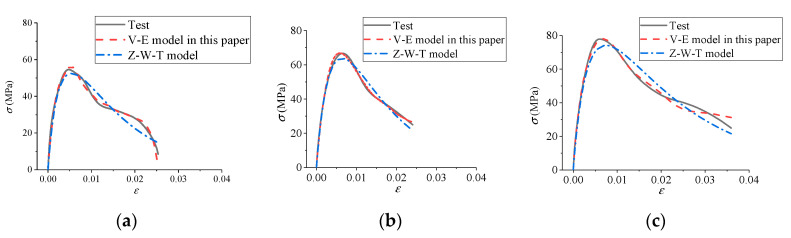
Comparison results of constitutive model curves under FT0. (**a**) 5.4 m/s; (**b**) 8.8 m/s; (**c**) 11.3 m/s.

**Figure 13:**
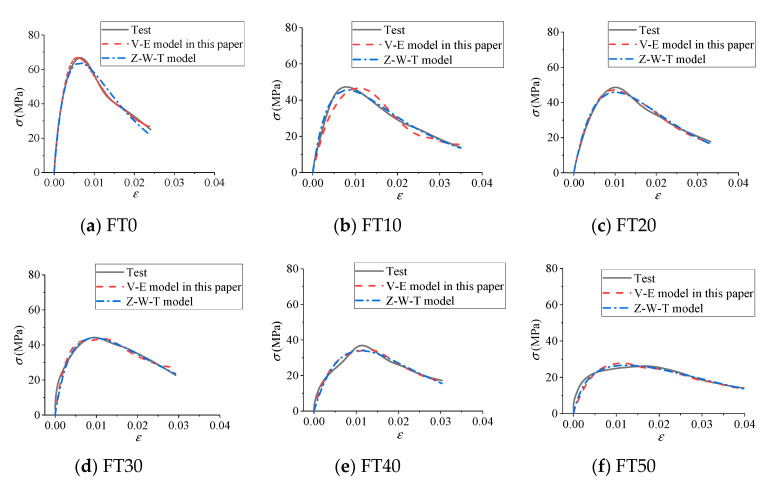
Comparison results of constitutive model curves under impact velocity of 8.8 m/s. (**a**) FT0; (**b**) FT10; (**c**) FT20; (**d**) FT30; (**e**) FT40; (**f**) FT50.

We took cases of FT0 with different impact velocities and an impact velocity of 8.8 m/s with different numbers of FT cycles as examples. Based on the test results, the visco-elastic constitutive model proposed in this paper and the Z-W-T model commonly used to describe the dynamic mechanical properties of concrete were respectively used for comparative study. The comparison results are shown in Figure 12 and Figure 13, and the values of different model parameters are shown in Table 2.

It can be seen from Table 2 and Figure 12 and Figure 13 that the visco-elastic constitutive model in this paper and the Z-W-T model were both in good agreement with the test results, indicating that the constitutive model deduced in this paper can effectively describe the dynamic mechanical properties of freeze–thawed concrete under impact load. In addition, the visco-elastic constitutive model in this paper had the advantages of fewer parameters and simpler expression compared with the Z-W-T model.

## 5. Damage Evolution Analysis

The damage of freeze–thawed concrete under impact load included two parts: freeze–thaw deterioration damage and impact stress damage. Among these, the freeze–thaw degradation damage is described by the freeze–thaw degradation factor ξ, and the stress fracture damage is characterized by the damage variable *D*.

As shown in Figure 8, the freeze–thaw degradation damage increased approximately linearly with the increase of FT cycles and decreased with the increase in impact velocity.

**Figure 14:**
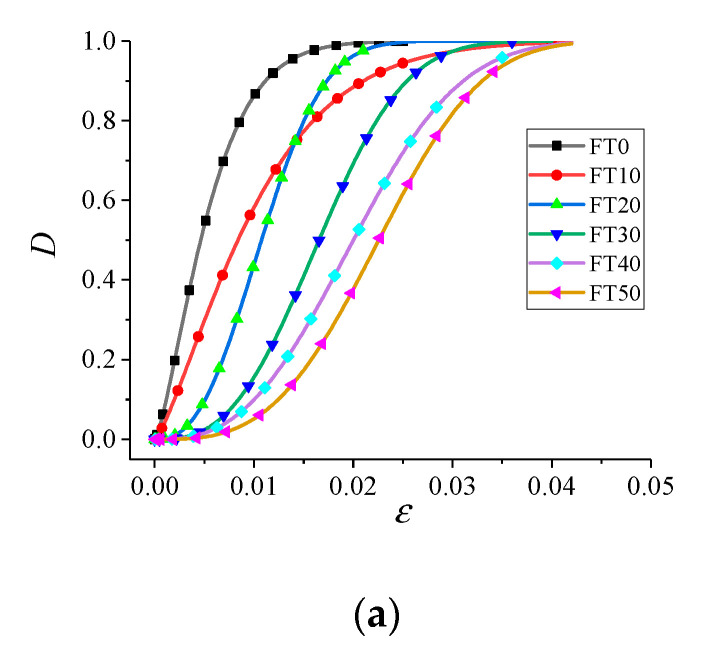

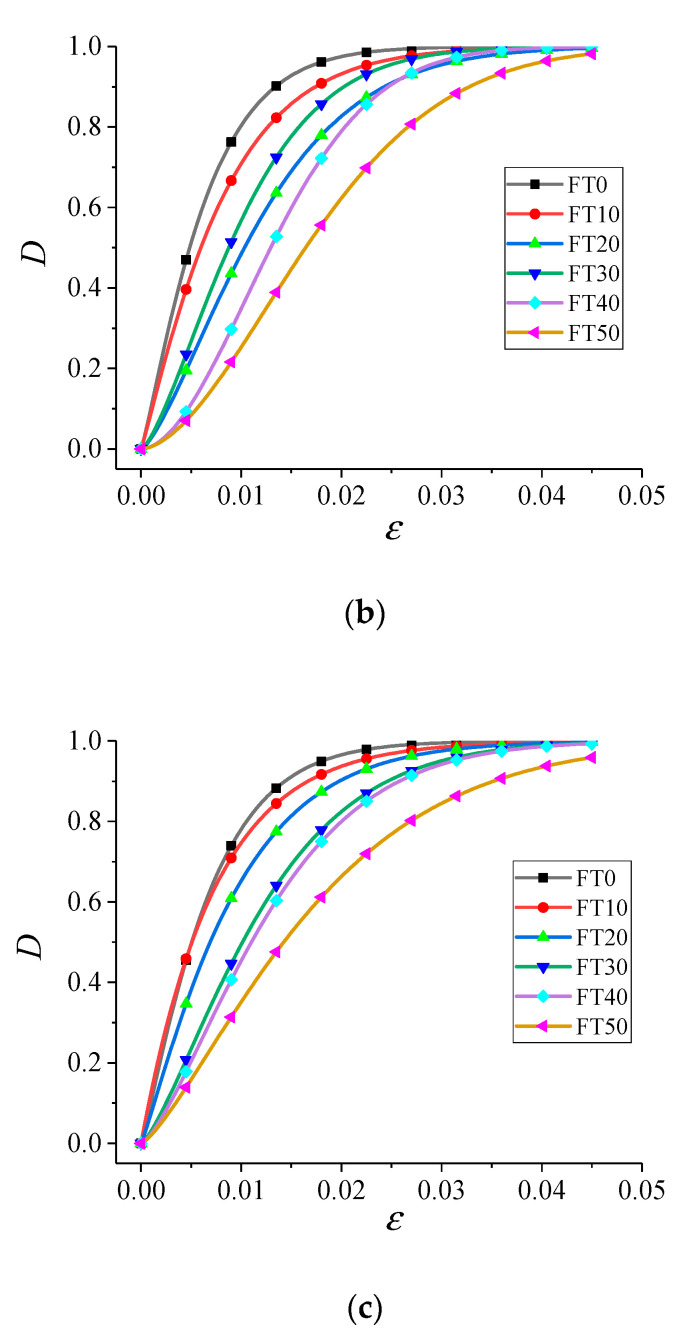
Damage evolution curve of freeze–thawed concrete. (**a**) 5.4 m/s; (**b**) 8.8 m/s; (**c**) 11.3 m/s.

According to Equation (10) and Table 2, the stress damage evolution process of concrete under different FT cycles and impact velocities can be obtained, as shown in Figure 14.

As shown in Figure 14, under different conditions, the stress damage evolution curves of concrete had roughly the same form. With the continuous accumulation of residual strain, the stress damage developed from fast to slow and finally tended to be stable.

Figure 14 also shows that, with the increase of FT cycles, the stress damage evolution path of concrete went backward, and the development rate of stress damage slowed down. Moreover, the greater the difference in the number of FT cycles, the greater the difference in the stress damage path. The specific differences in concrete stress damage path were mainly manifested as follows. When the cumulative strain was the same, with the increase of FT cycles, the stress damage *D* decreased gradually, and the slope of initial stress damage curves decreased constantly. This suggests that although the total damage of freeze–thawed concrete under impact load, which was the coupling result of freeze–thaw deterioration damage and impact stress damage, increased with the increase of the number of FT cycles, and as well as the freeze–thaw deterioration damage factor, while the stress damage value decreased. This was because the greater the number of FT cycles, the more serious the freeze–thaw deterioration; and the lower the residual energy consumption performance of the specimen was, the fewer the cracks generated by the specimen’s absorption of impact incident energy, namely, the smaller the stress damage was.

## 6. Conclusions

- The test results show that the freeze–thaw action and impact velocity have a significant influence on the dynamic fracture morphology, fracture block distribution and dynamic stress–strain curve of concrete, and the dynamic mechanical properties of freeze–thawed concrete are the coupling results of freeze–thaw deterioration effect and strain rate strengthening effect.
- By introducing the freeze–thaw deterioration damage factor and the stress damage variable, the dynamic visco-elastic damage constitutive model was deduced based on component combination theory. It can effectively describe the dynamic mechanical properties of freeze–thawed concrete, and has the characteristics of few parameters and good prediction accuracy.
- The stress damage evolution path of concrete goes backward with the increase of FT cycles and the development speed of stress damage gradually slows down. The greater the difference in FT cycles, the greater the difference of the stress damage path.

## Figures and Tables

**Table 1:** Fractal dimensions of crushing blocks under different conditions.

v	FT Cycles (Times)	Fitting Equation	χ
5.4	0	*y* = 1.1316*x* − 0.1577	1.8684
	10	*y* = 0.862*x* − 0.4298	2.138
	20	*y* = 0.882*x* − 0.0326	2.118
	30	*y* = 0.7487*x* − 0.3193	2.2513
	40	*y* = 0.8452*x* − 0.3875	2.1548
	50	*y* = 0.8188*x* − 0.3351	2.1812
8.8	0	*y* = 0.8875*x* − 0.4721	2.1125
	10	*y* = 0.6959*x* − 0.3039	2.3041
	20	*y* = 0.7724*x* − 0.3328	2.2276
	30	*y* = 0.7117*x* − 0.2985	2.2883
	40	*y* = 0.6958*x* − 0.3367	2.3042
	50	*y* = 0.7112*x* − 0.3101	2.2888
11.3	0	*y* = 0.7404*x* − 0.3232	2.2596
	10	*y* = 0.677*x* − 0.2953	2.323
	20	*y* = 0.6907*x* − 0.3081	2.3093
	30	y = 0.6244x − 0.2572	2.3756
	40	*y* = 0.6247*x* − 0.2556	2.3753
	50	*y* = 0.5689*x* − 0.2044	2.4311

Note: v stands for the impact velocity.

**Table 2:** Parameter inversion results of different constitutive models.

Models	FT Cycles (Times)	v **(m/s)**	m	α **(×10^−3^ s)**	η	$E_{0}$ **(GPa)**	$E_{1}$ **(GPa)**	$\varphi_{1}$ **(s)**	$E_{2}$ **(GPa)**	$\varphi_{2}$ **(×10^−6^ s)**	ξ	***R*** **^2^**
V-E model in this paper	0	5.4 8.8 11.3	1.36 1.18 1.15	0.95 2.68 3.30	0.180 0.070 0.072				15 18 21		0 0 0	0.98 0.99 0.99
	10	8.8	1.35	1.96	0.094				14		0.28	0.94
	20	8.8	1.52	1.55	0.034				9		0.29	0.99
	30	8.8	1.13	5.16	0.072				11		0.33	0.91
	40	8.8	1.86	0.44	0.041				6		0.43	0.96
	50	8.8	1.27	5.31	0.027				7		0.60	0.89
Z-W-T model	0	5.4 8.8 11.3	0.84 0.97 0.84	12.96 9.46 18.60		7.4 5.2 3.3	11.4 6.5 10.5	8.5 32.6 32.1	15 18 21	33.4 21.6 15.3		0.96 0.98 0.97
	10	8.8	1.05	12.10		1.0	1.3	94.4	14	30.6		0.99
	20	8.8	1.10	9.60		1.1	2.2	91.1	9	53.5		0.99
	30	8.8	1.09	10.73		1.1	3.3	69.0	11	19.7		0.90
	40	8.8	1.07	9.86		0.8	1.2	43.1	6	159.2		0.95
	50	8.8	1.04	15.57		0.9	2.0	18.3	7	11.0		0.93

## Abbreviations

**FT**	**Freeze–thaw**
SHPB	separated Hopkinson pressure bar
FT0	Freeze–thaw cycle carried out 0 times
FT10	Freeze–thaw cycle carried out 10 times
FT20	Freeze–thaw cycle carried out 20 times
FT30	Freeze–thaw cycle carried out 30 times
FT40	Freeze–thaw cycle carried out 40 times
FT50	Freeze–thaw cycle carried out 50 times
Z-W-T model	Zhu–Wang–Tang model
V-E model	visco-elastic constitutive model
